# Diabetes Mellitus

**Published:** 1913-03

**Authors:** William E. Fitch

**Affiliations:** Room 1002, 1600 Broadway, New York City


### CORRESPONDENCE.

### Diabetes Mellitus

I am undertaking an exhaustive research into the pathology, etiology and dieto-therapy of Diabetes Mellitus. I am very anxious to hear from every physician in the United States who has a case under treatment, or who has had any experience in the treatment of this malady. Von Noorden says "the best treatment for the diabetic is the *food* containing the *greatest* amount of *starch* which the patient can bear without harm." If any physician who reads this has similar or contrary experience, and would take the trouble to write me, I would esteem it a special privilege to hear from him, if only a postal card.

Kindly address

WILLIAM E. FITCH, M. D.,
Room 1002, 1600 Broadway,
New York City.

